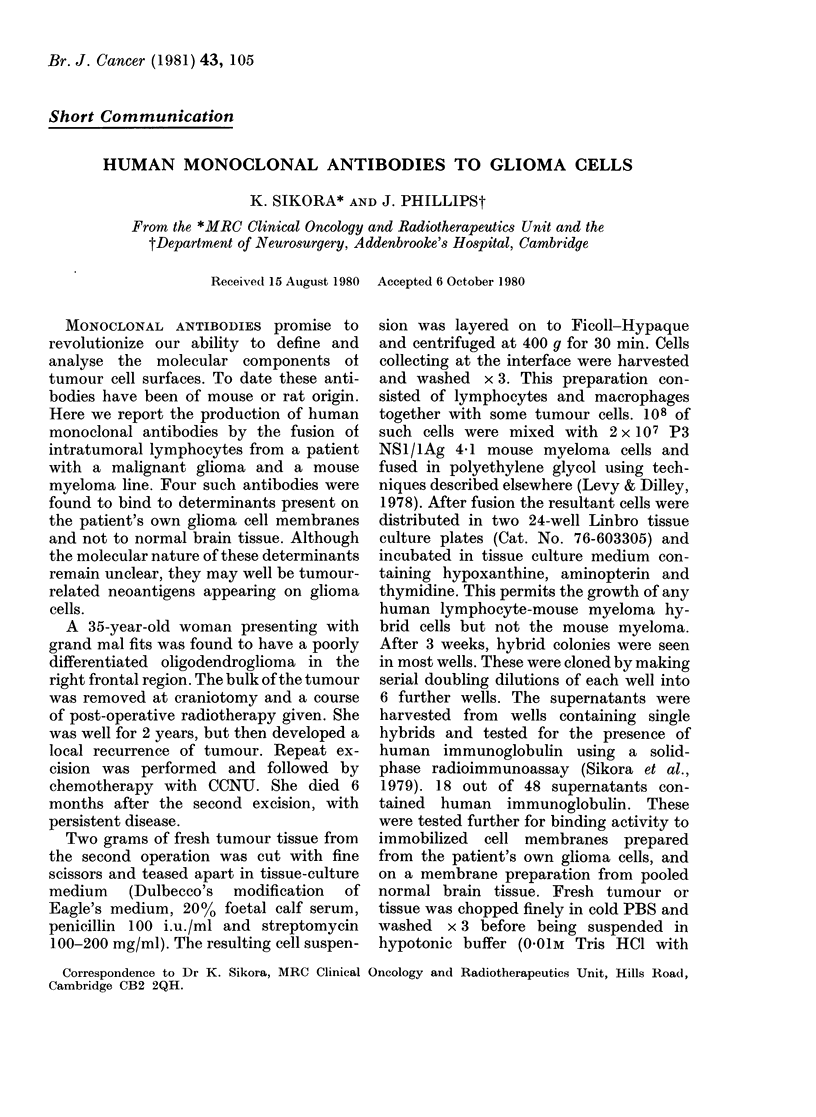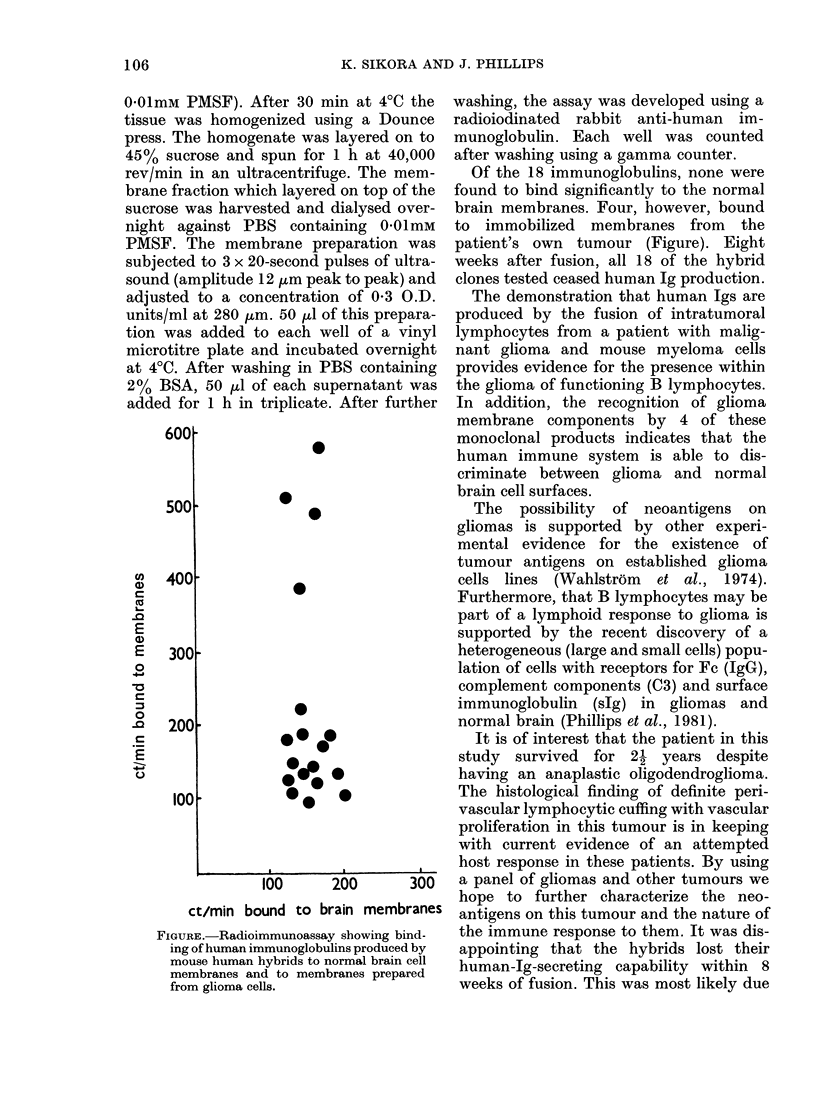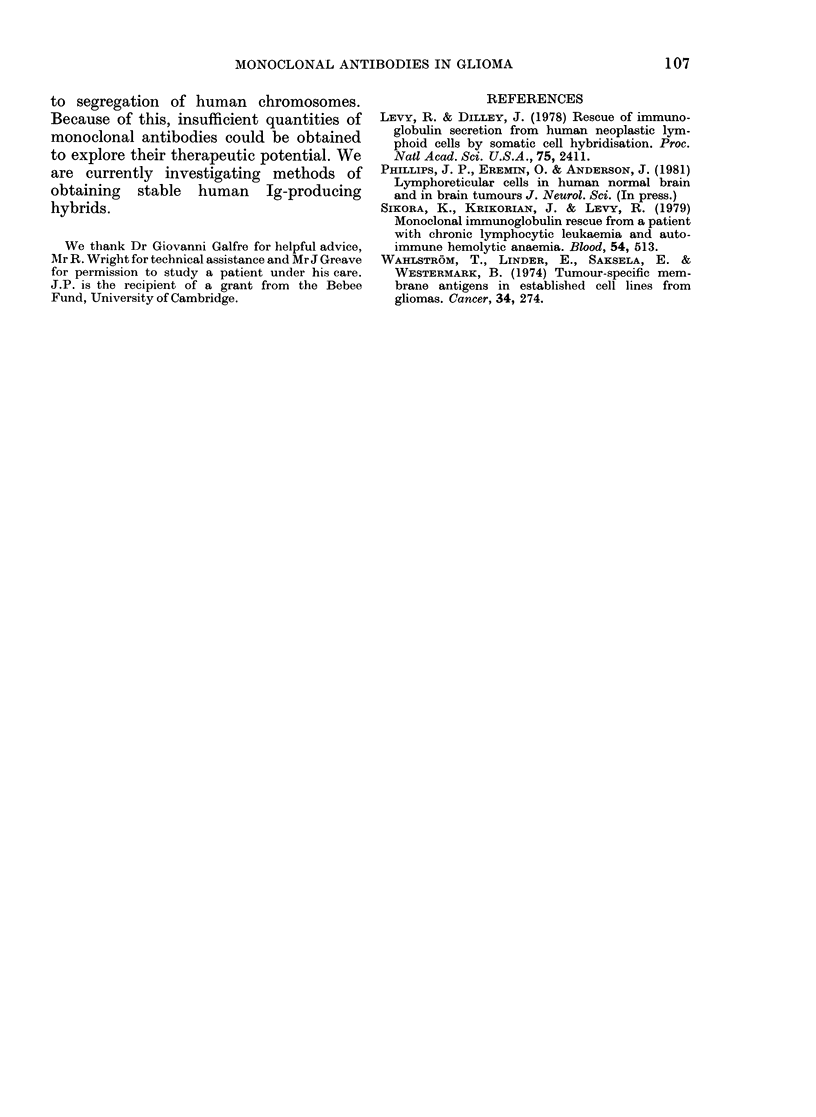# Human monoclonal antibodies to glioma cells.

**DOI:** 10.1038/bjc.1981.15

**Published:** 1981-01

**Authors:** K. Sikora, J. Phillips


*Br. J. Cancer* (1981) **43**, 105

***Short Communication***

### HUMAN MONOCLONAL ANTIBODIES TO GLIOMA CELLS

K. SIKORA* AND J. PHILLIPS†

*From the *MRC Clinical Oncology and Radiotherapeutics Unit and the †Department of Neurosurgery, Addenbrooke's Hospital, Cambridge*

Received 15 August 1980 Accepted 6 October 1980

MONOCLONAL ANTIBODIES promise to revolutionize our ability to define and analyse the molecular components of tumour cell surfaces. To date these antibodies have been of mouse or rat origin. Here we report the production of human monoclonal antibodies by the fusion of intratumoral lymphocytes from a patient with a malignant glioma and a mouse myeloma line. Four such antibodies were found to bind to determinants present on the patient's own glioma cell membranes and not to normal brain tissue. Although the molecular nature of these determinants remain unclear, they may well be tumour-related neoantigens appearing on glioma cells.

A 35-year-old woman presenting with grand mal fits was found to have a poorly differentiated oligodendroglioma in the right frontal region. The bulk of the tumour was removed at craniotomy and a course of post-operative radiotherapy given. She was well for 2 years, but then developed a local recurrence of tumour. Repeat excision was performed and followed by chemotherapy with CCNU. She died 6 months after the second excision, with persistent disease.

Two grams of fresh tumour tissue from the second operation was cut with fine scissors and teased apart in tissue-culture medium (Dulbecco's modification of Eagle's medium, 20% foetal calf serum, penicillin 100 i.u./ml and streptomycin 100–200 mg/ml). The resulting cell suspension was layered on to Ficoll–Hypaque and centrifuged at 400 *g* for 30 min. Cells collecting at the interface were harvested and washed ×3. This preparation consisted of lymphocytes and macrophages together with some tumour cells. $10^8$ of such cells were mixed with $2\times10^7$ P3 NS1/1Ag 4·1 mouse myeloma cells and fused in polyethylene glycol using techniques described elsewhere (Levy & Dilley, 1978). After fusion the resultant cells were distributed in two 24-well Linbro tissue culture plates (Cat. No. 76-603305) and incubated in tissue culture medium containing hypoxanthine, aminopterin and thymidine. This permits the growth of any human lymphocyte-mouse myeloma hybrid cells but not the mouse myeloma. After 3 weeks, hybrid colonies were seen in most wells. These were cloned by making serial doubling dilutions of each well into 6 further wells. The supernatants were harvested from wells containing single hybrids and tested for the presence of human immunoglobulin using a solid-phase radioimmunoassay (Sikora *et al.*, 1979). 18 out of 48 supernatants contained human immunoglobulin. These were tested further for binding activity to immobilized cell membranes prepared from the patient's own glioma cells, and on a membrane preparation from pooled normal brain tissue. Fresh tumour or tissue was chopped finely in cold PBS and washed ×3 before being suspended in hypotonic buffer (0·01M Tris HCl with

Correspondence to Dr K. Sikora, MRC Clinical Oncology and Radiotherapeutics Unit, Hills Road, Cambridge CB2 2QH.

0·01mM PMSF). After 30 min at 4°C the tissue was homogenized using a Dounce press. The homogenate was layered on to 45% sucrose and spun for 1 h at 40,000 rev/min in an ultracentrifuge. The membrane fraction which layered on top of the sucrose was harvested and dialysed overnight against PBS containing 0·01mM PMSF. The membrane preparation was subjected to 3 × 20-second pulses of ultrasound (amplitude 12 $\mu$m peak to peak) and adjusted to a concentration of 0·3 O.D. units/ml at 280 $\mu$m. 50 $\mu$l of this preparation was added to each well of a vinyl microtitre plate and incubated overnight at 4°C. After washing in PBS containing 2% BSA, 50 $\mu$l of each supernatant was added for 1 h in triplicate. After further

FIGURE.—Radioimmunoassay showing binding of human immunoglobulins produced by mouse human hybrids to normal brain cell membranes and to membranes prepared from glioma cells.

washing, the assay was developed using a radioiodinated rabbit anti-human immunoglobulin. Each well was counted after washing using a gamma counter.

Of the 18 immunoglobulins, none were found to bind significantly to the normal brain membranes. Four, however, bound to immobilized membranes from the patient's own tumour (Figure). Eight weeks after fusion, all 18 of the hybrid clones tested ceased human Ig production.

The demonstration that human Igs are produced by the fusion of intratumoral lymphocytes from a patient with malignant glioma and mouse myeloma cells provides evidence for the presence within the glioma of functioning B lymphocytes. In addition, the recognition of glioma membrane components by 4 of these monoclonal products indicates that the human immune system is able to discriminate between glioma and normal brain cell surfaces.

The possibility of neoantigens on gliomas is supported by other experimental evidence for the existence of tumour antigens on established glioma cells lines (Wahlström *et al.*, 1974). Furthermore, that B lymphocytes may be part of a lymphoid response to glioma is supported by the recent discovery of a heterogeneous (large and small cells) population of cells with receptors for Fc (IgG), complement components (C3) and surface immunoglobulin (sIg) in gliomas and normal brain (Phillips *et al.*, 1981).

It is of interest that the patient in this study survived for 2½ years despite having an anaplastic oligodendroglioma. The histological finding of definite perivascular lymphocytic cuffing with vascular proliferation in this tumour is in keeping with current evidence of an attempted host response in these patients. By using a panel of gliomas and other tumours we hope to further characterize the neoantigens on this tumour and the nature of the immune response to them. It was disappointing that the hybrids lost their human-Ig-secreting capability within 8 weeks of fusion. This was most likely due to segregation of human chromosomes. Because of this, insufficient quantities of monoclonal antibodies could be obtained to explore their therapeutic potential. We are currently investigating methods of obtaining stable human Ig-producing hybrids.

We thank Dr Giovanni Galfre for helpful advice, Mr R. Wright for technical assistance and Mr J Greave for permission to study a patient under his care. J.P. is the recipient of a grant from the Bebee Fund, University of Cambridge.

REFERENCES

LEVY, R. & DILLEY, J. (1978) Rescue of immunoglobulin secretion from human neoplastic lymphoid cells by somatic cell hybridisation. *Proc. Natl Acad. Sci. U.S.A.*, **75**, 2411.

PHILLIPS, J. P., EREMIN, O. & ANDERSON, J. (1981) Lymphoreticular cells in human normal brain and in brain tumours *J. Neurol. Sci.* (In press.)

SIKORA, K., KRIKORIAN, J. & LEVY, R. (1979) Monoclonal immunoglobulin rescue from a patient with chronic lymphocytic leukaemia and autoimmune hemolytic anaemia. *Blood*, **54**, 513.

WAHLSTRÖM, T., LINDER, E., SAKSELA, E. & WESTERMARK, B. (1974) Tumour-specific membrane antigens in established cell lines from gliomas. *Cancer*, **34**, 274.